# “Turn Up the Taste”: Assessing the Role of Taste Intensity and Emotion in Mediating Crossmodal Correspondences between Basic Tastes and Pitch

**DOI:** 10.1093/chemse/bjw007

**Published:** 2016-02-12

**Authors:** Qian Janice Wang, Sheila Wang, Charles Spence

**Affiliations:** Crossmodal Research Laboratory, Department of Experimental Psychology, University of Oxford, South Parks Road, Oxford OX1 3UD, UK

**Keywords:** crossmodal correspondences, emotion mediation, sound, taste intensity

## Abstract

People intuitively match basic tastes to sounds of different pitches, and the matches that they make tend to be consistent across individuals. It is, though, not altogether clear what governs such crossmodal mappings between taste and auditory pitch. Here, we assess whether variations in taste intensity influence the matching of taste to pitch as well as the role of emotion in mediating such crossmodal correspondences. Participants were presented with 5 basic tastants at 3 concentrations. In Experiment 1, the participants rated the tastants in terms of their emotional arousal and valence/pleasantness, and selected a musical note (from 19 possible pitches ranging from C2 to C8) and loudness that best matched each tastant. In Experiment 2, the participants made emotion ratings and note matches in separate blocks of trials, then made emotion ratings for all 19 notes. Overall, the results of the 2 experiments revealed that both taste quality and concentration exerted a significant effect on participants’ loudness selection, taste intensity rating, and valence and arousal ratings. Taste quality, not concentration levels, had a significant effect on participants’ choice of pitch, but a significant positive correlation was observed between individual perceived taste intensity and pitch choice. A significant and strong correlation was also demonstrated between participants’ valence assessments of tastants and their valence assessments of the best-matching musical notes. These results therefore provide evidence that: 1) pitch–taste correspondences are primarily influenced by taste quality, and to a lesser extent, by perceived intensity; and 2) such correspondences may be mediated by valence/pleasantness.

## Introduction

Researchers have recently started to reveal the extensive range of crossmodal correspondences that exist between sound and music on the one hand, and tastes, aromas, and flavors on the other (Belkin et al. 1997; Crisinel and Spence 2011; see Knöferle and Spence 2012, for a review). Soundtracks can be rated as sweet, salty, sour, or bitter depending on certain parameters of their composition, such as their pitch, articulation, loudness, etc. (e.g., Crisinel and Spence 2010a; Mesz et al. 2011; Knoeferle et al. 2015). For example, sweetness and sourness tend to correspond with sounds that are higher in pitch whereas bitterness corresponds with lower-pitched sounds instead (Crisinel and Spence 2009, 2010b). However, while the reliability of such crossmodal matches has become increasingly clear, the most appropriate explanation for such surprising crossmodal correspondences between seemingly unrelated stimuli in different sensory modalities has yet to be determined.

### Emotional mediation of crossmodal correspondences

One potential explanation for the crossmodal matching of sound with taste is in terms of emotional mediation. The suggestion here is that certain crossmodal correspondences may reflect the common emotional associations (such as pleasantness or arousal, see Collier 1996, for a reduction of emotion space to 2 dimensions) shared by the various stimuli involved. So, for instance, the correspondence between consonant musical harmony and sweetness (Wang and Spence 2016) may be attributable to people finding both stimuli pleasant. Such a hedonic matching account between seemingly unrelated stimuli presented in different sensory modalities explains, at least in part, color–music matching (Palmer et al. 2013), color–odor matching (Schifferstein and Tanudjaja 2004), and shape–taste matching (Velasco et al. 2016).

Relevant evidence pertaining to the case of crossmodal correspondences between audition and taste has, however, been limited to the pleasantness account. In an experiment designed to evaluate whether pleasantness mediates the crossmodal mapping between chocolate and sounds varying in their pitch and timbre (i.e., instrument type), Crisinel and Spence (2012b) reported that while the type of instrument sound chosen by their participants could be predicted on the basis of the pleasantness ratings they gave to the dark chocolate that they sampled, their choice of pitch could not. It should be kept in mind, however, that pleasantness constitutes but a single dimension of emotional space (Collier 1996). Importantly, to date, no one has yet examined the potential mediating role of emotional arousal in crossmodal correspondences between audition and taste.

### Measuring emotions

So far, we have used a model of valence and arousal for defining emotion, as opposed to a more categorical definition of basic emotions (Ekman 1992). In this study, we chose to address 2 dimensions of valence and arousal as opposed to, say, a set of 6 basic emotions (happiness, surprise, sadness, fear, anger, and disgust) because they are easier to measure, and because Wang et al. (2015) recently observed that participants uniquely associate each (albeit imagined) taste with different valence/arousal values (see also Cavanaugh et al., 2015, for evidence that different perceptual dimensions, including taste, can be used to differentiate emotions with different valence and arousal measurements). Using the valence/arousal definition of emotion also allows us to more easily test the pleasantness mediation theory that has been seen in other crossmodal correspondences, because pleasantness/valence is already one dimension that is being measured.

### Taste and emotion

In order to evaluate whether auditory–gustatory crossmodal associations are mediated by emotion, the emotional associations that people have with basic tastes must be established first. Although there has been much research on the influence of emotions on eating (see Macht 2008 and Singh 2014 for reviews) there is limited evidence on the emotions that may be evoked by (or associated with) basic taste stimuli. Robin et al. (2003) established emotional responses to the basic tastes based on autonomic nervous system parameters, which they then transcribed into 1 of 6 basic emotions (happiness, surprise, sadness, fear, anger, and disgust). According to their research, sweetness corresponded with “happiness” and “surprise,” bitterness was matched with “anger” and “disgust,” and sourness and saltiness elicited a variety of different emotions (Robin et al. 2003). In a study by Wang et al. (2015, Experiment 2), the taste words (bitter, salty, sour, and sweet) were shown to elicit different valence and arousal responses. For instance, sweetness had the highest valence and arousal ratings whereas bitterness had the lowest valence and arousal ratings. However, taste words don’t convey any information about how emotional responses might change as a function of differing taste intensity. To the best of our knowledge, Bredie et al. (2014) conducted the one and only study to have measured emotional responses to the basic tastes at multiple stimulus concentrations. However, the study was limited to facial expressions and self-reported pleasantness/valence ratings. In the present study, therefore, we collected emotional responses, both in terms of valence and arousal, for basic tastes solutions at multiple concentrations.

### Pitch and intensity

For whatever reason, pitch is one of the most frequently studied attributes in crossmodal correspondences involving the auditory modality. Researchers have, for instance, highlighted the existence of crossmodal associations with elevation (Ben-Artzi and Marks 1995; Evans and Treisman 2010; Parise et al. 2014), but also with brightness (Marks 1987), lightness (Marks 1987), size (Gallace and Spence 2006; Evans and Treisman 2010), aroma (Crisinel and Spence 2012a), and taste (Crisinel and Spence 2009, 2010a). In terms of taste–pitch matching, one drawback is that the sound–taste studies that have been conducted to date have utilized only single taste intensity. Of course, in real life, foods have tastes/flavors that vary widely in terms of their intensities. Here, we assessed whether variation in perceived taste intensity would influence such taste–pitch correspondences.

Previously, it has been shown that the method of magnitude matching between loudness and taste intensity, where participants choose a sound volume to match the perceived taste intensity, can highlight individual differences in taste perception and be used to establish a common basis for comparison across different participants (Gescheider 1997, pp. 285–287). In a study by Marks et al. (1988), taste intensity–volume matching was used to demonstrate that PROP (6-*n*-propylthiouracil) compounds were experienced differently by supertasters (who perceive a bitter taste) than nontasters (who don’t perceive any taste). Elsewhere, Bartoshuk used magnitude estimation to study the loss of taste perception in the elderly. They found that the elderly matched dilute tastes to louder sounds than young adults, possibly due to the elderly having a chronic background taste in the mouth (Bartoshuk et al. 1986).

In the studies reported here, participants were presented with basic taste solutions at 3 different concentrations, and were asked to choose a pitch that best matched each tastant. In addition to pitch, the participants were also asked to select loudness as a way of measuring subjective taste intensity in Experiment 1, and directly asked for their perceived taste intensity in Experiment 2.

## Experiment 1

The goals of Experiment 1 are 3-fold: 1) To gauge participants’ matching of basic taste solutions of different concentrations to both pitch and volume; 2) To examine how participants’ emotional rating of basic taste solutions changes with respect to increasing taste concentration; and 3) to evaluate the role of both valence/pleasantness and arousal in participants’ crossmodal associations between taste and pitch/volume.

### Methods

#### Participants

Thirty-three participants (19 women, 14 men) aged between 19 and 35 years (*M* = 24.03, standard deviation [SD] = 4.50) took part in the study. The participants gave their informed consent in writing, and reported no cold or other impairment of their senses of smell, taste, or hearing. The participants were recruited according to the Experimental Psychology Research Participation Scheme and Oxford Psychology Research Participant Database, and each participant was awarded either £5 or 2 course credits upon completion of the study. The study was approved by the Central University Research Ethics Committee of Oxford University (MSD-IDREC-C1-2014–205).

#### Taste stimuli

Approximately 10mL samples of bitter, sweet, sour, salty, and umami solutions were prepared, in 3 different concentrations (weak, medium, and strong). The intensity of all 5 tastes for each concentration level (4 = weak, 7 = medium, and 10 = strong) were matched according to taste intensity scales developed at the University of Minnesota in Saint Paul (M. Karalus, C. Pontet and Z. Vickers, unpublished data; see Table 1 for ingredients and concentrations of each of the taste solutions). The taste intensity scales were created by means of a 2-step process. First, a sourness scale was constructed by having the participants (*N* = 32) rate the intensity of 13 samples having different citric acid concentrations, dissolved in drinking water (Premium Waters). The best-fit regression line between concentration and intensity was used to determine citric acid concentrations for intensity values between 0 and 20. The sour scale was then used as a reference scale to create the bitter, sour, sweet, and umami scales (*N* = 20).

**Table 1.** Ingredients used to make each basic taste solution and the proportions used for each concentration level

Taste	Ingredient	Weak concentration (g/L)	Medium concentration (g/L)	Strong concentration (g/L)
Bitter	Caffeine	0.56	1.22	2.21
Salty	Salt (NaCl)	2.10	5.81	9.61
Sour	Citric acid	0.63	1.37	2.40
Sweet	Sugar	33.47	86.14	138.80
Umami	Monosodium glutamate (MSG)	0.94	18.73	44.95

The same solutions were used for both Experiment 1 and 2. All of the solutions were mixed with distilled water.

The solutions for this experiment were presented in clear 50mL clear plastic cups. The solutions themselves were both colorless and odorless.

#### Auditory stimuli

For the sound-matching task, there were 19 keys on the MIDI keyboard mapped from C2 to C8, with each consecutive key being 2 whole steps apart (so the keys were C2, E2, G#2, C3, E3, etc… up to C8). All of the notes used the Steinway piano synthesizer from Apple’s GarageBand software. The volume level was controlled by a dial with 7 radial markings around it. The volume at level 4, the middle marking, was approximately 75 dB. Each increase in level corresponded with approximately a 5 dB increase, with a maximum of approximately 90 dB at level 7. The participants wore HD-3030 stereo headphones during the sound-matching task.

#### Procedure

The experiment was conducted with participants sitting at a table in front of a computer monitor and a MIDI keyboard in an experimental booth. The experiment was programmed on the LimeSurvey online survey platform.

For the evaluation of the taste solutions, the participants were instructed to taste each sample by swirling the solution around their mouths for 3s, then expectorating. As a practice trial, participants were given a medium intensity solution of a random taste.

During the actual test, the participants were presented with 15 samples, one for each trial. For each trial, the participants were instructed to taste a particular sample, then rate their emotional response and pick a note (out of 19 choices) and volume setting on the MIDI keyboard that best matched the taste. The emotional responses were in terms of arousal and valence, both on a scale from −5 to +5. In order to help the participants make emotion ratings (especially arousal), they were presented with a 2D grid with valence on the *x* axis and arousal on the *y* axis. The four corners of the grid were anchored by excitement, relaxation, depression, and stress (Supplementary Appendix A). The order of all four questions for each trial, as well as the order of presentation of the taste stimuli, was randomized. The participants rinsed their mouths out with water between every trial. After all 15 samples had been presented, the participants were asked a series of post-trial questions about how much they enjoyed eating {bitter/salty/sour/sweet/umami}-tasting foods on a 5-point scale.

The entire study lasted for approximately 30min.

### Data analysis

Statistical software SPSS 23.0 (SPSS, Inc.) for Mac was used to analyze the results. A repeated measures analysis of variance (RM-ANOVA) with taste (bitter, salty, sour, sweet, and umami) and concentration (low, medium, and high) as the factors was conducted on participants’ choices of pitch and volume, as well as their ratings of valence and arousal. If the sphericity assumption was violated via the Mauchly Sphericity test, the degrees of freedom were adjusted using Greenhouse–Geisser or Huynh–Feldt corrections, as appropriate. All reported *P*-values in post hoc comparison tests have been Bonferroni corrected. To assess participants’ choice of pitch with respect to their individual assessment of intensity, we performed Pearson correlation analyses between pitch and volume ratings for each taste. Furthermore, the partial correlation coefficients between pitch, volume, valence, and arousal were calculated. A multiple linear regression was then performed in order to test whether participants’ rating of valence and arousal predicted their choice of pitch or volume. Finally, we analyzed participants’ self-reported liking for (bitter/salty/sour/sweet/umami) foods versus their valence ratings of the actual taste solutions using the Student’s *t*-test.

### Results

#### Auditory parameter choices

**Figure 1.**
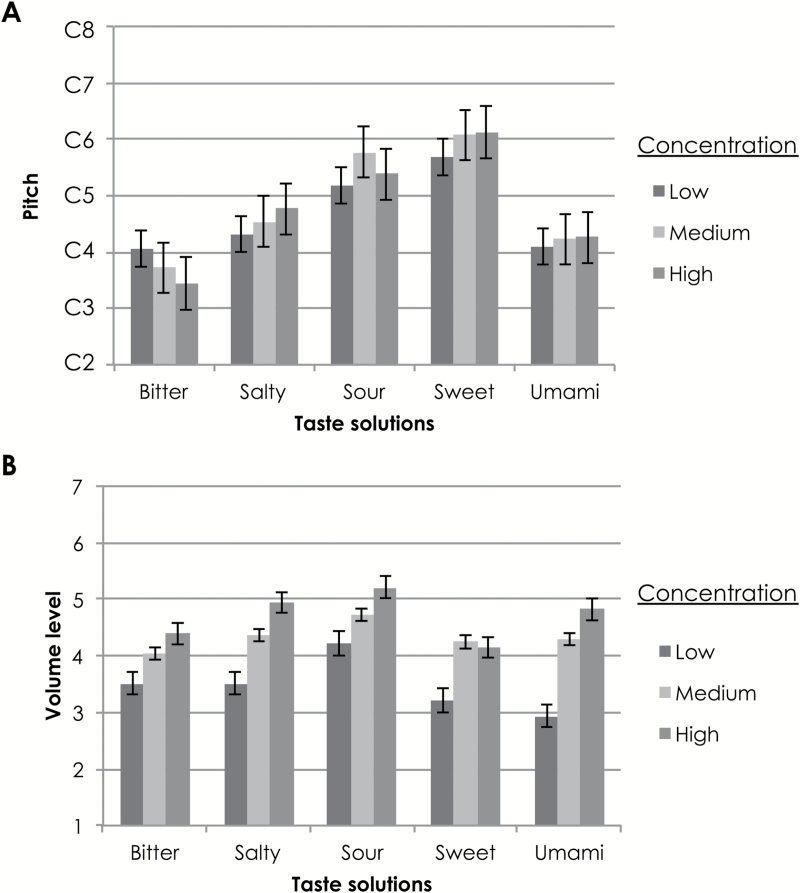
Mean values of participants’ choice of pitch (**A**) and volume/loudness (**B**) that best matches with various taste solutions in different concentrations in Experiment 1. Pitch is shown in musical notation, with differences between each line (e.g., from C2 to C3) being one octave (A). Volume levels ranged from 1 (60 dB) to 7 (90 dB) (B). The error bars denote the standard error of the mean.

The mean values of participants’ choices for pitch and volume to match with various taste solutions at different concentrations are shown in Figure 1.

RM-ANOVAs with taste (bitter, salty, sour, sweet, umami) and concentration (low, medium, high) as the factors were conducted on participants’ pitch and volume choices. In terms of participants’ choice of pitch (with scores ranging from 1, the lowest note, to 19, the highest note), a main effect of taste solution type was observed, *F*(4, 128) = 22.93, *P* < 0.0005, η^2^ = 0.42. Pairwise comparisons revealed that sweet (*M* = 12.88, SD = 3.27) and sour (*M* = 11.33, SD = 5.44) solutions were matched to a significantly higher pitch than the bitter (*M* = 6.22, SD = 4.36), salty (*M* = 8.63, SD = 4.23), and umami (*M* = 7.60, SD = 4.57) solutions, regardless of their concentration (*P* < 0.005 for all comparisons). In addition, the salty solution was matched to a sound having a significantly higher pitch than the bitter solution (*P* = 0.04).

In terms of participants’ choice of volume, a main effect of concentration levels was observed, *F*(2, 64) = 67.57, *P* < 0.005, η^2^ = 0.68, as predicted. Pairwise comparisons revealed that the low concentration solutions were matched to a significantly lower volume (*M* = 3.48, SD = 1.64) than the medium concentration (*M* = 4.33, SD = 1.47) solutions, which, in turn, were matched to a significantly lower volume than the high concentration solutions (*M* = 4.70, SD = 1.47; *P* < .05, for all comparisons). A significant main effect of taste solution type on volume ratings was also observed, *F*(3.43, 109.89) = 5.25, *P* = 0.001, η^2^ = 0.14, using Huynh–Feldt correction. Pairwise comparisons revealed that the sour solution (*M* = 4.72, SD = 1.38) was matched to a significantly higher volume than the salty (*M* = 4.27, SD = 1.41, *P* = 0.010), sweet (*M* = 3.87, SD = 1.64, *P* = 0.001), and umami (*M* = 4.02, SD = 1.97, *P* = 0.017) solutions.

Because volume can be interpreted as the participants’ rating of the perceived intensity of a given taste solution (Marks et al. 1988), we had reason to believe that it would more accurately reflect participants’ individual assessments of the intensity of the solutions than the concentration levels that we had prepared. Pearson correlations were therefore calculated between pitch and volume ratings to assess any relationships between participants’ perceived intensity and pitch choice. Overall, there was a significant positive correlation (*r*_495_ = 0.12, *P* = 0.006). More specifically, a significant positive relationship between perceived intensity (volume) and pitch choice was documented for the salty (*r*_99_ = 0.30, *P* = 0.002) and sweet (*r*_99_ = 0.31, *P* = 0.002) solutions. On the other hand, a significant negative relationship was observed for the bitter solutions (*r*_99_ = −0.20, *P* = 0.04). No relationship was observed for the sour (*r*_99_ = 0.15, *P* = 0.15) and umami (*r*_99_ = 0.17, *P* = 0.09) solutions. In other words, for salty and sweet tastes, increased taste intensity was associated with higher pitch, whereas for bitter tastes, increased taste intensity was associated with a lower pitch instead.

#### Emotion ratings

**Figure 2.**
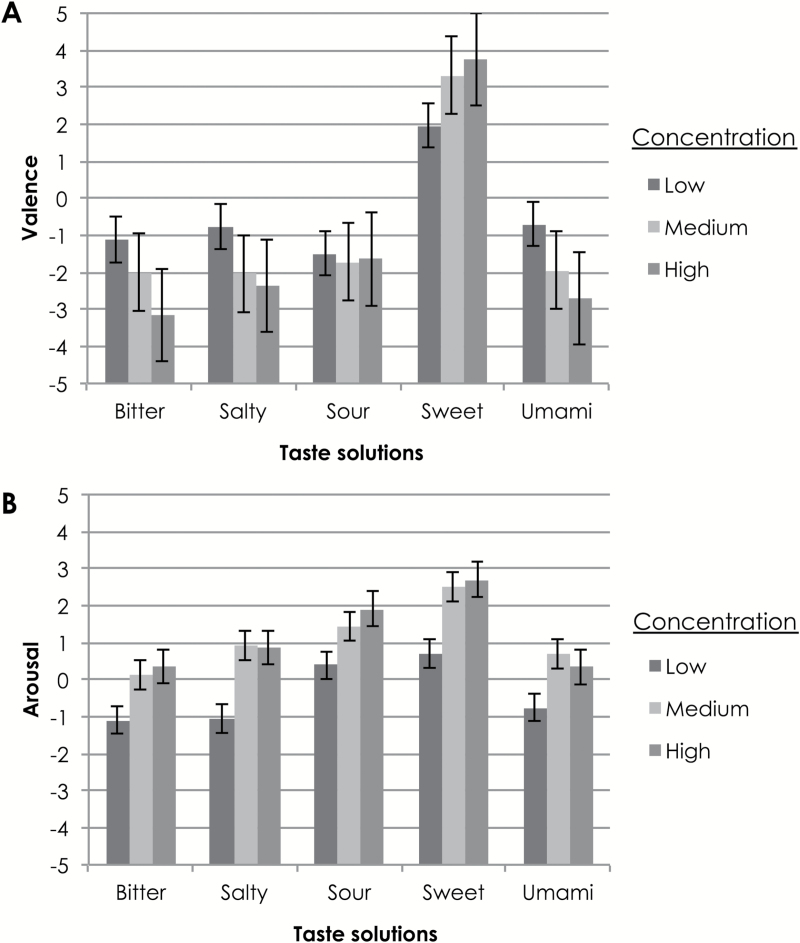
Mean values of participants’ rating of emotional valence/pleasantness (**A**) and arousal (**B**) associated with various taste solutions in different concentrations in Experiment 1. Valence is measured on a scale from −5 (extremely unpleasant) to +5 (extremely pleasant). Arousal is measured on a scale from −5 (not arousing at all) to +5 (extremely arousing). The error bars denote the standard error.

In terms of participants’ emotion ratings of various taste solutions, the mean ratings of valence and arousal are shown in Figure 2.

**Figure 3.**
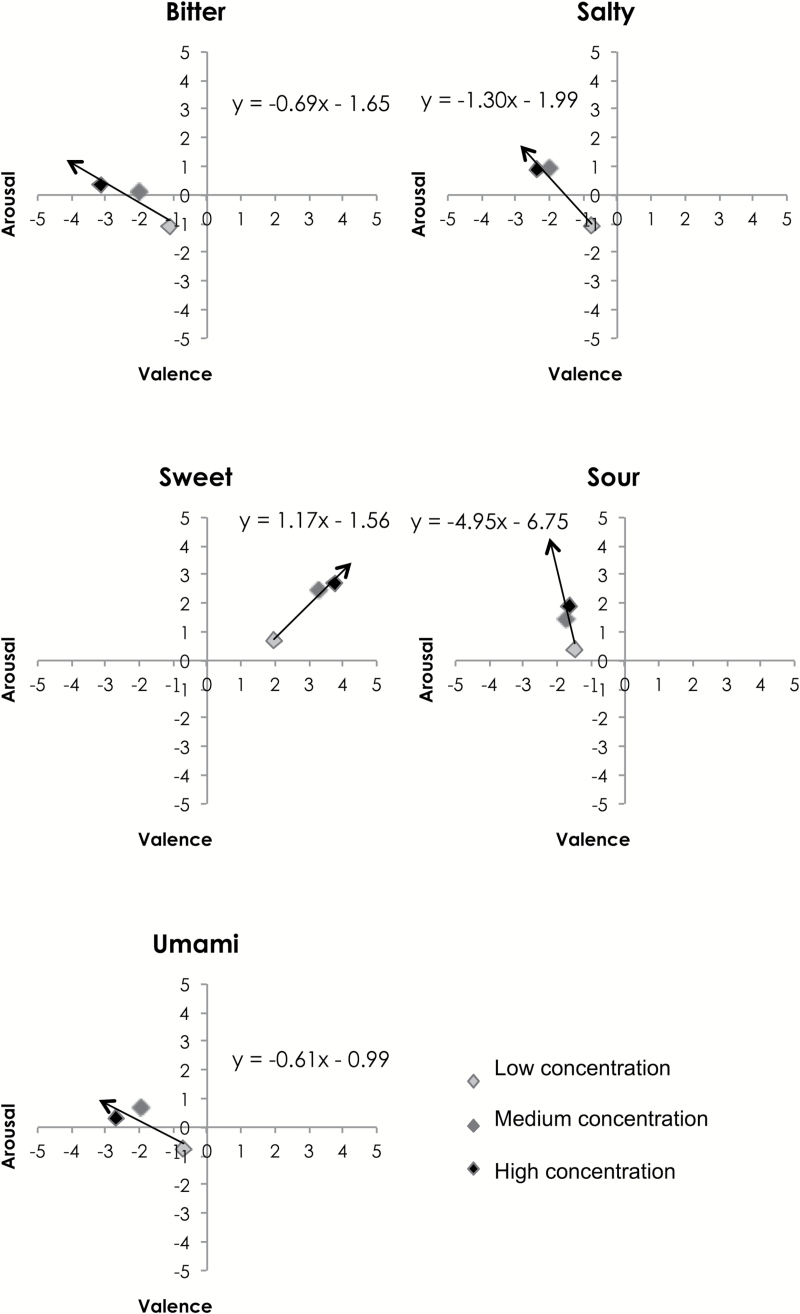
Mean values for participants’ emotion ratings of each taste solution in Experiment 1, plotted on a 2D valence-arousal graph. The horizontal valence axis ranges from +5 (extremely positive/pleasant) to −5 (extremely negative/unpleasant). The vertical arousal axis ranges from +5 (extremely arousing) to −5 (not arousing at all). For example, the top right corner (high valence and high arousal) would map to excitement, whereas the bottom left corner (low valence and low arousal) would map to depression. Different colours denote different concentrations. A trendline (with its equation shown) is plotted for each taste, with the direction of arrow indicating the direction of increasing concentration.

To get a better understanding of participants’ emotion ratings for the taste solutions having different concentrations, the average ratings of each taste were plotted for all 3 concentrations on the same graph, with trendlines shown as vectors pointing in the direction of increased concentration (Figure 3). Perhaps unsurprisingly, increasing concentration was associated with higher arousal levels. In general, bitter, salty, and umami solutions were rated as less pleasant and more arousing as the concentration increased. For the sour solutions, increases in arousal ratings were observed but little change in terms of pleasantness was seen. Only sweet solutions *increased* in terms of their pleasantness as the concentration of the solutions increased. Presumably, we did not reach the level of sweetness at which participants began to dislike the solution (Giovanni and Pangborn 1983).

RM-ANOVA tests with taste and concentration as factors were conducted on participants’ ratings of valence and arousal. In terms of participants’ choice of valence, a main effect of taste solution type was observed, *F*(2.78, 88.80) = 65.16, *P* < 0.0005, η^2^ = 0.67, using Greenhouse–Geisser correction. Pairwise comparisons revealed, unsurprisingly, that the sweet solution was matched to significantly higher valence (*M* = 3.01, SD = 1.79) than the bitter (*M* = −2.09, SD = 2.01), salty (*M* = −1.72, SD = 1.97), sour (*M* = −1.62, SD = 2.67), or umami (*M* = −1.78, SD = 2.59) solutions, regardless of the concentration at which the tastant was presented (*P* < 0.0005 for all comparisons). A significant main effect of concentration was also observed, *F*(1.66, 53.01) = 8.18, *P* = 0.002, η^2^ = 0.20, using Huynh–Feldt correction. Pairwise comparisons revealed that the low concentration solutions (*M* = −0.42, SD = 2.26) were rated significantly higher on the valence scale (i.e., as more pleasant) than the medium (*M* = −0.88, SD = 3.04, *P* = 0.013) and the high concentration solutions (*M* = −1.22, SD = 3.39, *P* = 0.001). An interaction effect was also observed between the taste of the solution and its concentration, *F*(5.72, 183.08) = 7.44, *P* < 0.0005, η^2^ = 0.19. In particular, as concentration increased, valence ratings were higher for sweet solutions, but lower for bitter, salty, and umami solutions. Specifically, low concentration sweet solutions (*M* = 1.97, SD = 1.96) were rated as significantly less pleasant than medium (*M* = 3.30, SD = 1.78, *P* = 0.14) and high concentration sweet solutions (*M* = 3.76, SD = 1.00, *P* < 0.0005). High concentration bitter solutions (*M* = −3.15, SD = 1.75) were rated as significantly less pleasant than low (*M* = −1.12, SD = 2.00, *P* < 0.0005) and medium concentration bitter solutions (*M* = −2.00, SD = 1.77, *P* = 0.027). The high concentration umami solution (*M* = −2.70, SD = 2.74) was rated as significantly less pleasant than the low concentration umami solution (*M* = −0.70, SD = 1.59, *P* = 0.003). Finally, the low concentration salty solution (*M* = −0.76, SD = 1.50) was rated as more pleasant than the medium (*M* = −2.03, SD = 1.70, *P* = 0.003) and high concentration salty solutions (*M* = −2.36, SD = 2.29, *P* < 0.0005).

In terms of participants’ choice of arousal, a main effect of concentration levels was observed, *F*(1.30, 41.51) = 18.97, *P* < 0.005, η^2^ = 0.37, using Greenhouse–Geisser correction. Pairwise comparisons revealed that the low concentration (*M* = −0.36, SD = 2.42) solutions were rated as significantly less arousing than the medium (M = 1.13, SD = 2.69) and high concentration solutions (*M* = 1.24, SD = 3.40; *P* < 0.0005 for all comparisons). A main effect of taste solution type on arousal ratings was also observed, *F*(2.84, 90.86) = 7.49, *P* < 0.0005, η^2^ = 0.19, using Greenhouse–Geisser correction. Pairwise comparisons revealed that the sweet solution (*M* = 1.96, SD = 2.13) was rated as significantly more arousing than the bitter (*M* = −0.20, SD = 3.08, *P* = .003), salty (*M* = 0.24, SD = 2.81, *P* = 0.049) and umami (*M* = 0.09, SD = 3.06, *P* = 0.005) solutions; in addition, the sour solution (*M* = 1.25, SD = 3.06) was rated as significantly more arousing than the salty solution (*P* = 0.036) and almost as more arousing than the bitter solution (*P* = 0.063).

Pearson correlations between participants’ choices of auditory attributes (pitch and volume) and their emotion ratings (valence and arousal) were then computed over all taste solutions. For pitch, positive moderate correlations between pitch and valence (*r*_495_ = 0.32) and between pitch and arousal (*r*_495_ = 0.28) were found. For volume, a moderate negative correlation with valence (*r*_495_ = −0.25) and a moderate positive correlation with arousal (*r*_495_ = 0.21) was documented. All correlations were significant (*P* < 0.0005).

As many of the variables are correlated, partial correlation coefficients were calculated in order to control for the effect of possible third variables (*r*_495_ = 0.31 between pitch and valence, *r*_495_ = 0.17 between pitch and arousal, *r*_495_ = −0.35 between volume and valence, and *r*_495_ = 0.25, between volume and arousal). All coefficients remained significant (*P* < 0.0005).

A multiple linear regression was used to test whether participants’ rating of the valence and arousal of a given taste solution significantly predicted their choice of matching pitch or volume. The results of the regression indicated that for pitch, valence (β = 0.27, *P* < 0.0005) and arousal (β = 0.22, *P* < 0.0005) accounted for 14.8% of the variance, *R* ^2^ = 0.148, *F*(2, 492) = 42.87, *P* < 0.0005. For volume, valence (β = −0.32, *P* < 0.0005) and arousal (β = 0.29, *P* < 0.0005) accounted for 14.0% of the variance, *R* ^2^ = 0.140, *F*(2, 492) = 40.21, *P* < 0.0005. As valence and arousal are correlated (*r*_495_ = 0.26, *P* < 0.0005), we also analyzed them independently. Valence alone accounted for 10.5% of the variance in pitch choice [β = 0.32, *P* < 0.0005, *F*(1, 494) = 32.67, *P* < 0.0005] and 6.2% of the variance in volume choice [β = −0.25, *P* < 0.0005, *F*(1, 494) = 57.76, *P* < 0.0005]. Arousal alone accounted for 8.1% of the variance in pitch choice [β = 0.28, *P* < 0.0005, *F*(1, 494) = 43.35, *P* < 0.0005] and 4.3% of the variance in volume choice [β = 0.21, *P* < 0.0005, *F*(1, 494) = 22.11, *P* < .0005].

Analysis of the post-experiment questions revealed that the average self-reported liking was 1.94 (SD = 1.20) for bitter foods, 3.27 (SD = 1.01) for salty foods, 2.82 (SD = 1.13) for sour foods, 4.15 (SD = 0.87) for sweet foods, and 4.12 (SD = 0.86) for umami foods. We compared this data with the average valence ratings of each taste solution (averaged over all concentration levels) and found that ratings were significantly different for salty [*t*(98) = 7.69, *P* < .0005], sour [*t*(98) = 3.90, *P* < .0005], and umami [*t*(98) = 14.76, *P* < .0005] tastes.

Finally, some individual differences were observed in terms of taste preferences. We identified 6 out of the 33 participants as potential supertasters based on the criteria that they matched the weak concentration bitter solution with a volume level of at least 6 out of 7 (see Bartoshuk et al. 1994; Hall et al. 1975, for evidence that sensitivity to PTC predicts sensitivity to caffeine, which was used here for the bitter solutions). Interestingly, these “bitter-sensitive” participants (*M* = 3.89, SD = 4.52) matched bitter solutions to significantly lower pitches than the remainder of the participants (*M* = 6.74, SD = 4.18), *t*(97) = 2.58, *P* = 0.011. They also made higher valence ratings (*M* = 0.11, SD = 2.74) for sour solutions than the rest of the participants (*M* = −2.00, SD = 2.51), *t*(97) = −3.18, *P* = 0.002.

### Discussion

The results of Experiment 1 demonstrate the relationship between taste concentration, auditory pitch, volume, and both emotional valence and arousal. Differences in taste quality (bitter/salty/sour/sweet/umami) exerted a significant influence over the participants’ choice of pitch and loudness as well as their ratings of arousal and valence. With increasing taste concentration, the participants chose louder sounds (as predicted), as well as higher arousal and lower valence ratings (except for sweet tastes, where increasing taste concentration increased valence ratings). For sweet and salty tastes, the choice of pitch was positively correlated with perceived taste intensity (as represented by loudness) whereas for bitter tastes, pitch choice was negatively correlated.

For those ratings where the concentration had a significant main effect (namely valence, arousal, and loudness), it is interesting to note that we did not find any significant differences between medium and high concentration for valence and arousal ratings, although we did find a significant difference between medium and high concentrations for loudness choices. Perhaps participants categorize taste concentrations as either high or low when it comes to expressing different degrees of valence and arousal, but when it comes to matching volumes, participants are better able to express differences. (Alternatively, however, perhaps making an intensity match between taste concentration and volume, both physical properties, is easier than matching taste concentration with more abstract ideas such as valence and/or arousal).

It is of interest for future research to note that people’s self-reported liking for foods of a given taste may be different from their actual experience of the taste. Sour, salty, and umami were all liked significantly more as taste words (in the form of “sour/salty/umami-tasting foods”) when actually consumed (albeit in liquid form). This means that for experimental studies involving hedonic measurements, it may be more appropriate to have one’s participants consume real foods rather than using taste words (for instance, Velasco et al. 2014, showed a relationship between roundness/angularity ratings and liking for actual tastes, but not for taste words).

Overall, in Experiment 1, we observed a possible impact of both emotional valence and arousal on taste-note matching. However, because the participants chose pitch and volume and made emotion ratings in the same experimental block, it is possible that their choice of sound attributes were based on their emotion ratings. Therefore, in Experiment 2, the experimental design was changed so that the participants now made sound matches and emotion ratings in different blocks of trials. In addition, we have so far only made indirect assessments of taste intensity based on participants’ volume selection. To double check our assertion about loudness acting as a proxy for subjective intensity, participants rated the taste intensity of the solutions directly in Experiment 2 instead of selecting a loudness setting.

## Experiment 2

In this study, we addressed the possible concern that the participants used their emotion ratings to select notes by separating them into separate blocks. We also had the participants make emotion ratings for the musical notes in order to make a more comprehensive emotional mediation analysis.

### Methods

#### Participants

33 participants (23 women, 10 men) aged between 18 and 30 years (*M* = 21.67, SD = 3.28) took part in the study. The participants gave their informed consent in writing, and reported no cold or other impairment of their senses of smell, taste, or hearing. The participants were recruited in a similar manner as Experiment 1, and each participant was awarded either £7 or 4 course credits upon completion of the study. The study was approved by the Central University Research Ethics Committee of Oxford University (MSD-IDREC-C1-2014–205).

#### Taste stimuli

The taste stimuli were identical to those used in Experiment 1.

#### Auditory stimuli

The musical note selection setup was identical to that used in Experiment 1. In the music note emotion evaluation block, the participants listened to 2-s sound recordings of the same 19 notes available on the MIDI keyboard, recorded using the Steinway piano synthesizer from Apple’s Garageband software.

#### Procedure

Experiment 2 differs from Experiment 1 by separating sound-matching and emotion-rating tasks into different blocks of trials. First, the participants tasted each sample in a random order, and had to choose a musical note (same selection process as Experiment 1) that best matched the taste. On the next screen, the participants had to rate the perceived intensity of the sample they just tasted. The participants rinsed their mouths with water between every trial. After the first block, participants were given a piece of cracker to eat to cleanse their palate while taking a 10-min break. In the second block, the participants first practiced making valence/arousal ratings in response to images. Next, they tasted the samples again in a random order and made valence/arousal ratings for each sample, rinsing their mouths with water in between trials. Finally, in the third block, the participants listened to 2-s sound clips of all 19 music notes, in a random order, and made valence/arousal ratings for each note.

The entire study lasted for approximately 45min.

### Data analysis

Statistical software SPSS 23.0 (SPSS, Inc.) for Mac was used to analyze the results. A RM-ANOVA with taste (bitter, salty, sour, sweet, umami) and concentration (low, medium, high) as the factors was conducted on participants’ choice of pitch as well as ratings of intensity, valence, and arousal. If the sphericity assumption was violated via the Mauchly sphericity test, the degrees of freedom were adjusted using Greenhouse–Geisser or Huynh–Feldt corrections, as appropriate. All reported *P* values in post hoc comparison tests have been Bonferroni corrected. To assess participants’ choice of pitch with respect to their individual assessment of intensity, we performed Pearson correlation analyses between pitch and volume ratings for each taste. Furthermore, we calculated the partial correlation coefficients between pitch, volume, valence, and arousal. Because we collected emotion data relating to both tastants and musical notes, we calculated the correlation between the emotion ratings of tastants and the emotion ratings of the music notes best matched with each tastant (see Palmer et al. 2013, for an example of the same method).

### Results

#### Auditory parameter choice

**Figure 4.**
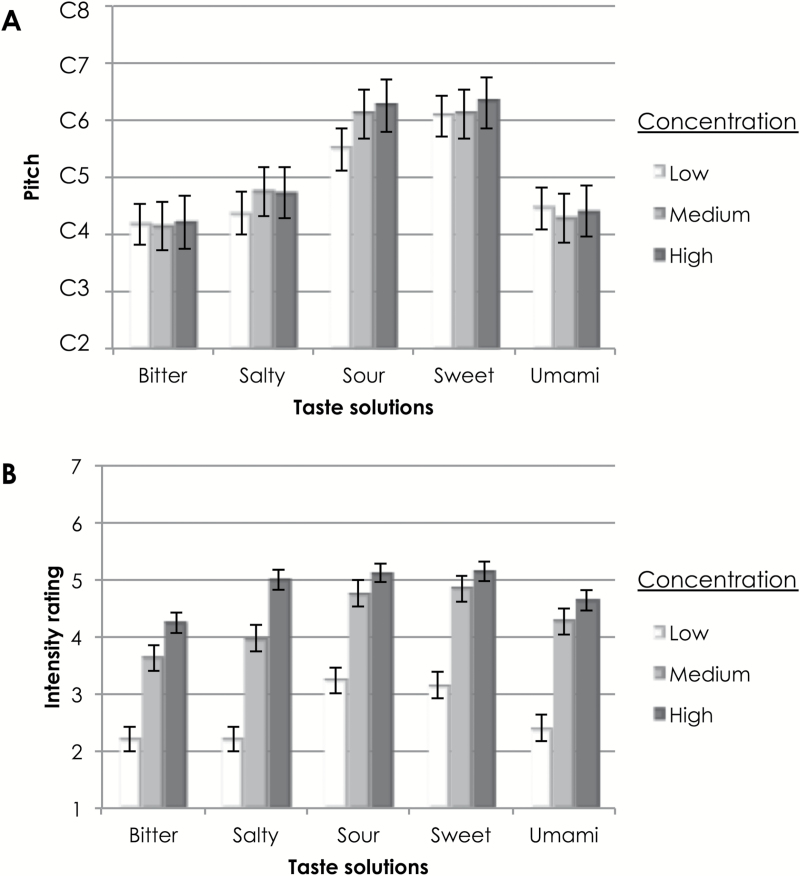
Mean values of participants’ choice of best-matching pitch (**A**) and taste intensity (**B**) for various taste solutions in different concentrations in Experiment 2. Pitch is shown in musical notation, with differences between each line (e.g., from C2 to C3) being one octave (A). Intensity rating was on a scale from 1 to 7 (B). The error bars denote standard error.

The mean values of participants’ choices for pitch to match with various taste solutions at different concentrations are shown in Figure 4A.

An RM-ANOVA with taste (bitter, salty, sour, sweet, umami) and concentration (low, medium, high) as the factors was conducted on participants’ choices of pitch. A main effect of taste solution type was observed, *F*(4, 128) = 25.43, *P* < 0.0005, η^2^ = 0.44. Pairwise comparisons revealed that sweet (*M* = 13.47, SE = 0.55) and sour (*M* = 12.84, SE = 0.55) solutions were matched to a significantly higher pitch than the bitter (*M* = 7.54, SE = 0.58), salty (*M* = 8.83, SD = 0.53), and umami (*M* = 8.14, SD = 0.45) solutions, regardless of their concentration (*P* < 0.005 for all comparisons).

To validate that the participants did indeed perceive the solutions at different concentrations, we assessed the participants’ rating of intensity (see Figure 4B for mean rating values). As predicted, a main effect of concentration was observed, *F*(2, 64) = 144.39, *P* < 0.0005, η^2^ = 0.82. Pairwise comparisons revealed that the low concentration solutions were rated as significantly less intense (*M* = 3.48, SD = 1.64) than the medium concentration (*M* = 4.33, SD = 1.47) solutions, which, in turn, were rated as being less intense than the high concentration solutions (*M* = 4.70, SD = 1.47; *P* < 0.05 for all comparisons). A significant main effect of taste solution type on intensity ratings was also observed, *F*(3.58, 114.70) = 6.25, *P* < 0.0005, η^2^ = 0.16, using Huynh–Feldt correction. Pairwise comparisons revealed that the sweet solution (*M* = 4.38, SE = 0.20) was overall significantly more intense than the bitter (*M* = 3.36, SE = 0.24) and salty (*M* = 3.73, SE = 0.14) solutions. In addition, the sour solution (*M* = 4.37, SE = 0.18) was also significantly more intense than the bitter solution. This suggests that participants in fact did not perceive the solutions to be at equal intensity, as claimed by Karalus et al. (unpublished data).

As in Experiment 1, any relationship between participants’ perceived taste intensity and pitch choice was assessed by calculating Pearson correlations between pitch and intensity ratings of each taste. Unlike in Experiment 1, we used participants’ actual taste intensity ratings and not a secondary measure (e.g., volume) that might reflect perceived taste intensity. Across all tastes, a significant weak positive correlation between taste intensity and choice of pitch was shown, *r*_495_ = 0.12, *P* = 0.006. More specifically, a significant positive relationship between perceived intensity and pitch choice was observed for the sour solution (*r*_99_ = 0.23, *P* = 0.023). On the other hand, a significant negative relationship was observed for the umami solution (*r*_99_ = −0.25, *P* = 0.011). In other words, for sour solutions, increased perceived taste intensity was associated with a higher pitch, whereas for the umami solutions, increased perceived taste intensity was associated with a lower pitch instead. No significant correlations were observed for other tastes (for bitter, *r*_99_ = −0.15, *P* = 0.14; for salty, *r*_99_ = 0.15, *P* = 0.15; for sweet, *r*_99_ = 0.15, *P* = 0.15).

#### Emotion ratings

**Figure 5.**
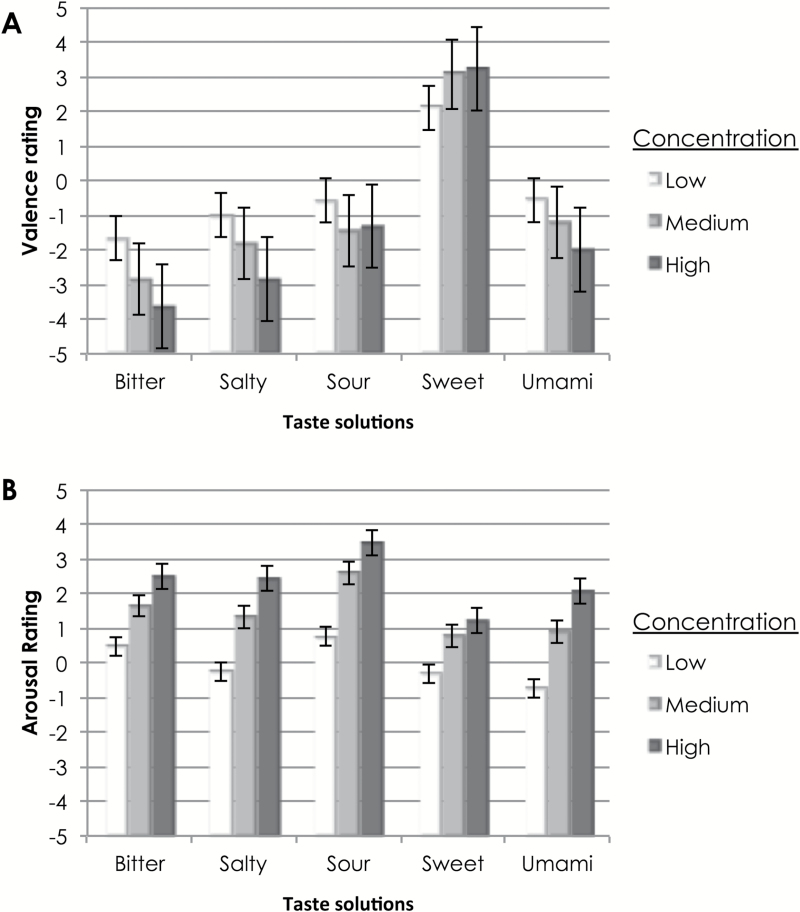
Mean values of participants’ rating of emotional valence/pleasantness (**A**) and arousal (**B**) associated with various taste solutions in different concentrations in Experiment 2. Valence is measured on a scale from −5 (extremely unpleasant) to 5 (extremely pleasant). Arousal is measured on a scale from −5 (not arousing at all) to 5 (extremely arousing). The error bars denote the standard error.

In terms of participants’ emotion ratings of various taste solutions, the mean ratings of valence and arousal are shown in Figure 5.

RM-ANOVA tests with taste and concentration as factors were conducted on participants’ ratings of valence and arousal. In terms of participants’ choice of valence, a main effect of taste solution type was observed, *F*(3.43, 109.67) = 62.55, *P* < 0.0005, η^2^ = 0.66, using Huynh–Feldt correction. Unsurprisingly, pairwise comparisons revealed that the sweet solution was matched to a significantly higher valence (*M* = 2.82, SE = 0.24) than the bitter (*M* = −2.72, SD = 0.19), salty (*M* = −1.89, SD = 0.28), sour (*M* = −1.11, SD = 0.34), or umami (*M* = −1.25, SD = 0.32) solutions, regardless of the concentration at which the tastant was presented (*P* < 0.0005 for all comparisons). In addition, the bitter solutions were significantly less pleasant than the sour, sweet, and umami solutions. A significant main effect of concentration was also observed, *F*(2, 64) = 19.17, *P* < 0.0005, η^2^ = .38. Pairwise comparisons revealed that the low concentration solutions (*M* = −0.33, SE = 0.14) were rated significantly higher on the valence scale (i.e., as more pleasant) than the medium (*M* = −0.85, SE = 0.18, *P* = 0.003) and the high concentration solutions (*M* = −1.31, SE = 0.19, *P* < 0.0005); in addition, the medium concentration solutions were more pleasant than the high concentration solutions (*P* = 0.015). An interaction effect was also observed between the taste of the solution and its concentration, *F*(8, 256) = 8.68, *P* < 0.0005, η^2^ = 0.21. In particular, as concentration increased, valence ratings were higher for sweet solutions, but lower for bitter, salty, and umami solutions. Specifically, low concentration sweet solutions (*M* = 2.12, SE = 0.25) were rated as significantly less pleasant than the medium (*M* = 3.10, SE = 0.29, *P* = 0.006) and high concentration (*M* = 3.24, SE = 0.33, *P* = 0.002) sweet solutions. High concentration bitter solutions (*M* = −3.64, SE = 0.23) were rated as significantly less pleasant than the low (*M* = −1.67, SE = 2.7, *P* < 0.0005) and medium concentration (*M* = −2.85, SE = 0.25, *P* = 0.043) bitter solutions; the medium concentration bitter solution was also rated as less pleasant than the low concentration solution (*P* < 0.0005). The high concentration salty solution (*M* = −2.85, SE = 0.32) was rated as less pleasant than both the low (*M* = −1.00, SE = 0.25, *P* < 0.0005) and medium concentration (*M* = −1.82, SE = 0.44, *P* = 0.01) salty solutions. The medium concentration sour solution (*M* = −1.46, SE = 0.35) was less pleasant than the low concentration sour solution (*M* = −0.58, SE = 0.37, *P* = 0.009). Finally, the high concentration umami solution (*M* = −2.00, SE = 0.46) was rated as significantly less pleasant than the low (M = −0.55, SE = 0.24, *P* = 0.006) and medium (M = −1.21, SE = 0.42, *P* = 0.045) concentration umami solutions.

In terms of participants’ choice of arousal, a main effect of concentration was observed, *F*(2, 64) = 51.41, *P* < 0.0005, η^2^ = 0.62. Pairwise comparisons revealed that the low concentration (*M* = −0.006, SE = 0.17) solutions were rated as significantly less arousing than the medium (*M* = 1.46, SE = 0.22) and high concentration solutions (*M* = 2.33, SE = 0.20), and that medium concentration solutions were less arousing than high concentration solutions (*P* < 0.0005 for all comparisons). A main effect of taste solution type was also observed on arousal ratings, *F*(3.64, 116.55) = 8.90, *P* < 0.0005, η^2^ = 0.22, using Huynh–Feldt correction. Pairwise comparisons revealed that the sour solution (*M* = 2.27, SE = 0.21) was rated as significantly more arousing than the salty (*M* = 1.18, SE = 0.25, *P* = 0.002), sweet (*M* = 0.57, SE = 0.29, *P* < 0.0005) and umami (*M* = 0.75, SE = .25, *P* < 0.0005) solutions.

Pearson correlations between participants’ choices of pitch, intensity ratings, and their emotion ratings (valence and arousal) were then computed over all taste solutions. As many of the variables are correlated, partial correlation coefficients were calculated in order to control for the effect of possible third variables. For pitch, positive moderate correlations between pitch and valence (*r*_495_ = 0.39, *P* < 0.0005) and between pitch and arousal (*r*_495_ = 0.13, *P* = 0.004) were found. For intensity, moderate positive correlation was observed between intensity rating and arousal (*r*_495_ = 0.32, *P* < 0.0005) but there was no significant correlation between intensity rating and valence (*r*_495_ = 0.034, *P* = 0.45). In addition, a significant correlation was observed between arousal and valence, *r*_495_ = *−*0.37, *P* < 0.0005.

As in Experiment 1, a multiple linear regression was used to test whether participants’ rating of the valence and arousal of a given taste solution significantly predicted their choice of matching pitch. The results of the regression indicated that for pitch, valence (β = 0.43, *P* < 0.0005) and arousal (β = 0.17, *P* < 0.0005) accounted for 15.6% of the variance, *R* ^2^ = 0.156, *F*(2, 492) = 45.53, *P* < 0.0005.

Analyzing the emotional factors separately, valence alone accounted for 13% of the variance in pitch choice, β = 0.36, *P* < 0.0005, *F*(1, 494) = 73.96, *P* < 0.0005. By contrast, arousal alone did not predict the participants’ choice of pitch.

Extending the results of Experiment 1, we also collected data about participants’ emotion ratings of all 19 musical notes from C2 to C8. Mean values of valence and arousal ratings for the notes can be seen in Supplementary Appendix B. It is worth noting that valence ratings make an inverted U-shape, where pitches that are at the low (approximately pitches 1–5, or C2 to E3) or high (approximately pitches 16–19, or C7 to C8) end are more unpleasant than the notes in the middle. Inversely, arousal ratings make a U-shape, where pitches at the low and high end of the keyboard are more arousing than the notes in the middle.

**Figure 6.**
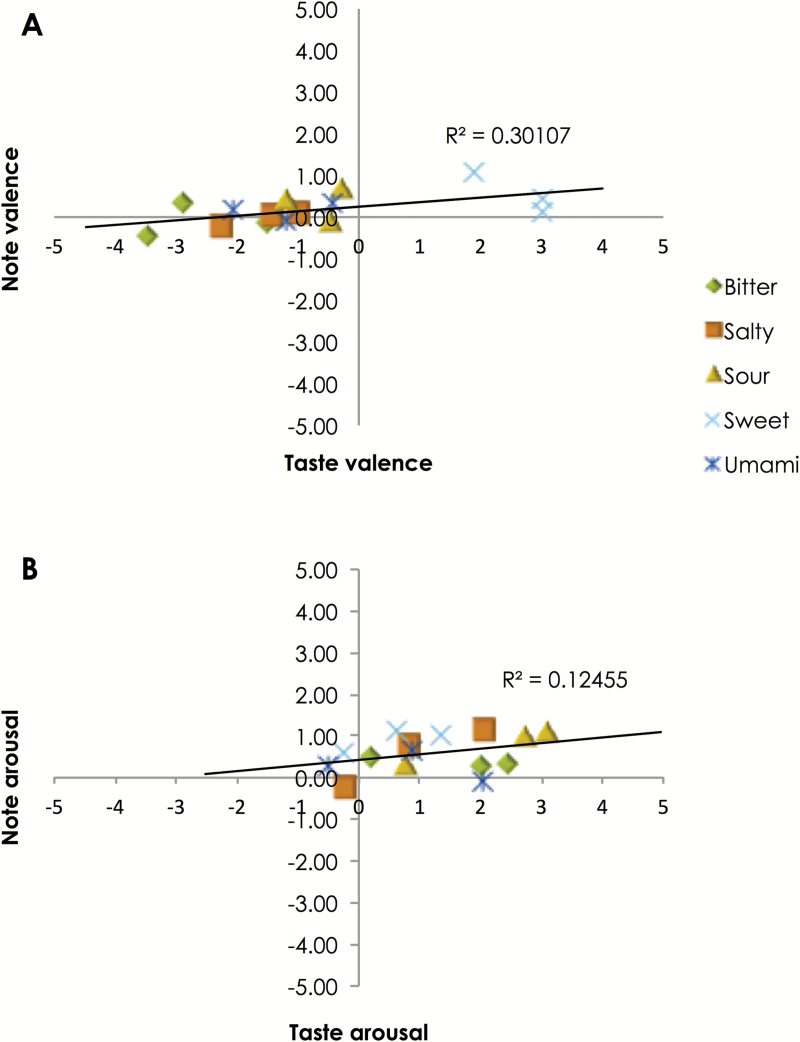
Scatterplots and correlations between the emotional ratings of the 15 taste solutions (*x* axis) and the emotional associations of the pitch chosen as most matching with them (*y* axis), for the 2 emotional dimensions valence (**A**) and arousal (**B**).

Using the method from Palmer et al. (2013), the correlation between the emotion ratings of each taste and the emotion ratings of the note that it matched with were examined (Figure 6). There were 2 separate analyses for valence and arousal. In terms of valence, there was a significant positive correlation between valence ratings for each taste sample and the note that best matched the sample, *r* = 0.55, *P* = 0.034. However, no significant correlation between arousal ratings of taste samples and matching notes were found, *r* = 0.35, *P* = 0.195.

### Discussion

As in Experiment 1, differences in taste quality (bitter/salty/sour/sweet/umami) exerted a significant influence over the participants’ choice of pitch and perceived taste intensity as well as their ratings of arousal and valence. With increasing taste concentration, the participants rated solutions as more intense (as expected) as well as giving higher arousal ratings and lower valence ratings (except for sweet tastes, where increasing taste concentration increased valence ratings). In addition, a weak positive correlation was observed between perceived taste intensity and pitch choice, suggesting that taste intensity does affect taste–pitch matching, but to a less extent than taste quality.

In Experiment 2, the major change was to move emotion ratings to a separate block, after participants have already made their pitch selection for each tastant. This was to rule out the possibility that participants did not base their pitch selection on their emotion ratings, which was possible in Experiment 1. Nevertheless, significant correlations between pitch choices and emotion ratings were still observed across blocks, thus showing that the relationship between pitch choice and emotion rating is a genuine one.

Given the additional information of emotion ratings for musical notes, we were able to perform a more complete emotional mediation analysis by comparing emotion ratings of each taste solution with the emotion ratings of the musical note that it best matched with. This is the first time, to the best of our knowledge, where emotional response to individual musical notes have been measured and used to analyze emotion mediation effects in crossmodal correspondences involving pitch. We did not observe a significant correlation in the emotional dimension of arousal, but we did observe a significant positive correlation in the dimension of valence. In other words, the more pleasant the taste, the more pleasant the musical note chosen to match with the taste. It is worth noting here that this conclusion is different from Crisinel and Spence’s (2012b) results, where no relationship was found between the pitch that was chosen and the pleasantness of the dark chocolate that was sampled. However, Crisinel and Spence’s (2012b) analysis was limited to a single food item and the participants’ emotional response to pitch was not measured.

## General discussion

So, should researchers take taste intensity into account when studying crossmodal correspondences between sound and taste? In general, the intensity of the tastants would seem to be correlated with attributes that also have magnitude measurements, such as higher loudness selection and higher arousal ratings. Such correspondences can be categorized as examples of prothetic (magnitude-related) matching (see Spence 2011 for a review). In addition, we also found a correlation between self-reported intensity ratings and pitch. Together, these findings suggest that taste intensity should be carefully controlled in future experiments.

The idea that pitch is associated with emotional valence as well as perceived taste intensity sheds new light on possible hypotheses behind the crossmodal matching of pitch and taste. First, it would seem that, as in color–music and color–odour correspondences, hedonic matching plays a role in taste–pitch correspondences. However, hedonic matching does not explain the overall positive association between perceived taste intensity and pitch, because higher taste intensity is generally associated with lower valence, but higher pitch is associated with higher valence. One other theory is a statistical learning account based on the innate orofacial gestures that infants make in response to sweet (outwards and upwards tongue positions) versus bitter tastes (outwards and downwards tongue positions) and the associated utterances (Scherer 1986; Knöferle and Spence 2012). With respect to intensity, one can imagine that foods with more intense flavors would lead people to make more energetic sounds, which are higher in frequency (Scherer 1986).

Yet another theory behind pitch-taste matching is metaphorical or semantic mapping, where people may use the same language (e.g., sharp, delicate, heavy) in order to describe both pitch and taste. This theory might encompass both the valence mediation results and the intensity–pitch correlation we have observed thus far. For instance, sweet tastes and high pitch might both be described as delicate, with positive connotations, whereas bitter tastes and low pitch might both be described as heavy, with negative connotations. With regard to the correlation between taste intensity and pitch, a possible semantic explanation is that both high intensity and high pitch might be described as “sharp.” An interesting follow-up experiment would be to repeat the pitch-matching exercise with foods having easily identifiable basic tastes (bitter/salty/sour/sweet/umami) but with differing textures. For instance, imagine 2 sets of taste solutions of the same concentration, one mixed with water and the other thickened with starch. If the metaphorical matching theory holds, then one would expect the tastant with a heavier body to be matched to a lower pitch than the tastants with a lighter body.

In closing, given this relationship between pitch choice and emotion ratings, predictions can certainly be made about how participants match novel or more complex tastes and flavors to pitch. So, for instance, mint gum, which is arguably both pleasant and intensely flavored, would be mapped to a relatively high pitch. Taking another example, those who enjoy eating spicy foods might map spicy foods to a higher pitch, whereas those who don’t enjoy spicy foods might map them to a lower pitch. We look forward to future studies where such predictions involving more complex foods and personal preferences will be assessed.

## Supplementary material

Supplementary material can be found at http://www.chemse.oxfordjournals.org/

## Funding

This work was supported by the AHRC grant entitled “Rethinking the senses” (AH/L007053/1 to C.S.).

## Supplementary Material

Supplementary Data
