# Supplementary material for: “Turn Up the Taste”: Assessing the Role of Taste Intensity and Emotion in Mediating Crossmodal Correspondences between Basic Tastes and Pitch
Source: Chem Senses. 2016 Feb 12;41(4):345–56. doi: 10.1093/chemse/bjw007 (PMC4840871; doi:10.1093/chemse/bjw007)
Supplement: Supplementary Data [file supp_41_4_345__index.html]

“Turn Up the Taste”: Assessing the Role of Taste Intensity and Emotion in Mediating Crossmodal Correspondences between Basic Tastes and Pitch — “Turn Up the Taste”: Assessing the Role of Taste Intensity and Emotion in Mediating Crossmodal Correspondences between Basic Tastes and Pitch — Supplementary Data 

# “Turn Up the Taste”: Assessing the Role of Taste Intensity and Emotion in Mediating Crossmodal Correspondences between Basic Tastes and Pitch

## Supplementary Data

Data files

- Supplementary Data - Supplementary Data
- Supplementary Data - Supplementary Data
