# Supplementary figures and images for: “Turn Up the Taste”: Assessing the Role of Taste Intensity and Emotion in Mediating Crossmodal Correspondences between Basic Tastes and Pitch
Source: Chem Senses. 2016 Feb 12;41(4):345–56. doi: 10.1093/chemse/bjw007 (PMC4840871; doi:10.1093/chemse/bjw007)

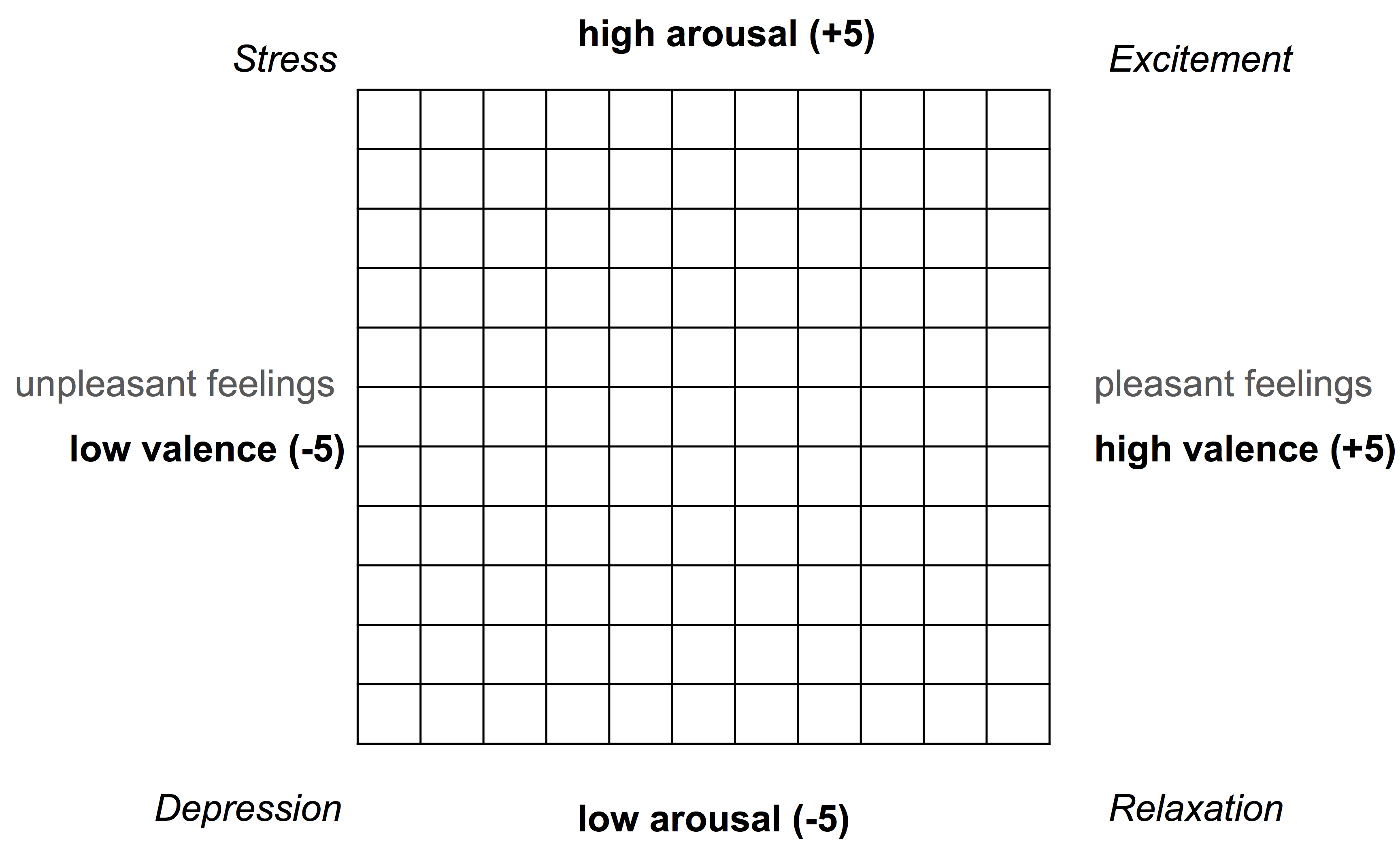

Supplement: Supplementary Data [file supp_bjw007_appendix_a.tif]

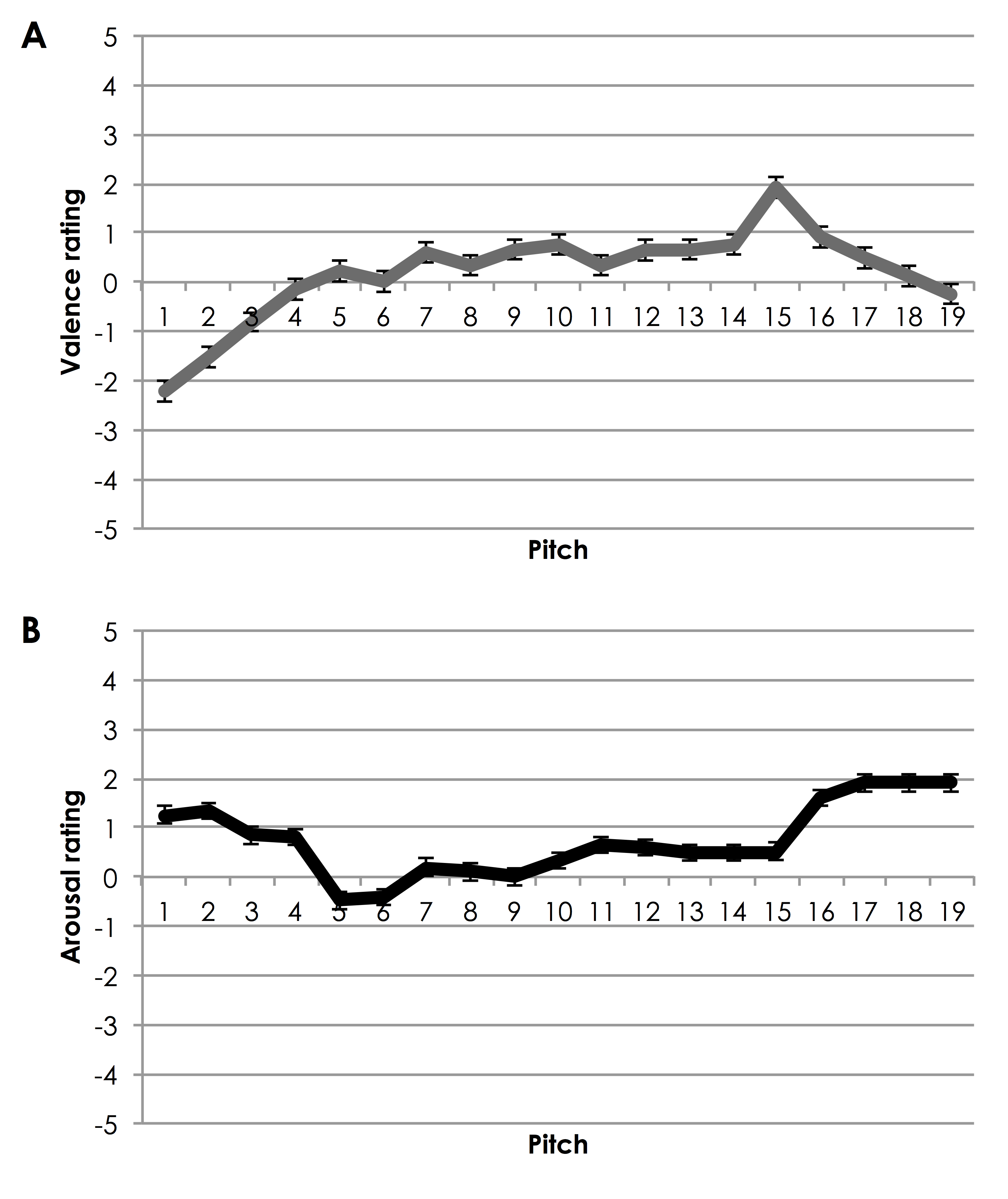

Supplement: Supplementary Data [file supp_bjw007_appendix_b.tif]
